# Harbor porpoise losing its edge: Genetic time series suggests a rapid population decline in Iberian waters over the last 30 years

**DOI:** 10.1002/ece3.10819

**Published:** 2023-12-11

**Authors:** Yacine Ben Chehida, Tjibbe Stelwagen, Jeroen P. A. Hoekendijk, Marisa Ferreira, Catarina Eira, Andreia Torres‐Pereira, Lidia Nicolau, Julie Thumloup, Michael C. Fontaine

**Affiliations:** ^1^ Groningen Institute for Evolutionary Life Sciences (GELIFES) University of Groningen Groningen The Netherlands; ^2^ Department of Biology University of York York UK; ^3^ Ecology and Evolutionary Biology, School of Biosciences University of Sheffield Sheffield UK; ^4^ BirdEyes, Centre for Global Ecological Change at the Faculties of Science & Engineering and Campus Fryslân University of Groningen Leeuwarden The Netherlands; ^5^ Department of Coastal Systems, NIOZ Royal Netherlands Institute for Sea Research Utrecht University Texel The Netherlands; ^6^ Wageningen University & Research Centre Wageningen The Netherlands; ^7^ MATB‐Portuguese Wildlife Society (SPVS) Figueira da Foz Portugal; ^8^ ECOMARE, Universidade de Aveiro Aveiro Portugal; ^9^ Centre for Environmental and Marine Studies CESAM University of Aveiro Aveiro Portugal; ^10^ MIVEGEC, Univ. Montpellier, CNRS, IRD Montpellier France

**Keywords:** approximate Bayesian computation, bycatch, cetacean conservation genetics, genetic diversity, genetic time series, population genetic modeling

## Abstract

Impact of climate change is expected to be especially noticeable at the edges of a species' distribution, where they meet suboptimal habitat conditions. In Mauritania and Iberia, two genetically differentiated populations of harbor porpoises (*Phocoena phocoena*) form an ecotype adapted to local upwelling conditions and distinct from other ecotypes further north on the NE Atlantic continental shelf and in the Black Sea. By analyzing the evolution of mitochondrial genetic variation in the Iberian population between two temporal cohorts (1990–2002 vs. 2012–2015), we report a substantial decrease in genetic diversity. Phylogenetic analyses including neighboring populations identified two porpoises in southern Iberia carrying a divergent haplotype closely related to those from the Mauritanian population, yet forming a distinct lineage. This suggests that Iberian porpoises may not be as isolated as previously thought, indicating possible dispersion from Mauritania or an unknown population in between, but none from the northern ecotype. Demo‐genetic scenario testing by approximate Bayesian computation showed that the rapid decline in the Iberian mitochondrial diversity was not simply due to the genetic drift of a small population, but models support instead a substantial decline in effective population size, possibly resulting from environmental stochasticity, prey depletion, or acute fishery bycatches. These results illustrate the value of genetics time series to inform demographic trends and emphasize the urgent need for conservation measures to ensure the viability of this small harbor porpoise population in Iberian waters.

## Graphical abstract

The Iberian harbor porpoises form a small and semi‐isolated population belonging to a distinctive ecotype adapted to the upwelling system in the southern European Atlantic waters. Here, we analyzed the mitochondrial genetic variation for a sampling spanning over the past 30 years and reported a dramatic decline in diversity. Simulation‐based statistical scenario testing demonstrates that this loss of diversity corresponds to an ancient expansion, followed by a dramatic decline in the Iberian population size over the last three generations. These results lineup with field observations and call for urgent conservation actions.

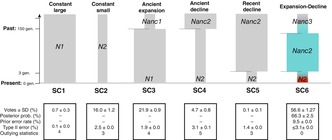

## 1 INTRODUCTION

The impact of climate change on species is expected to be especially noticeable at their distribution edges, where species meet the limits of their ecological tolerance. Marginal populations of temperate species at the rear (warm) distribution edge are often considered more susceptible to warming than central populations because of the warmer ambient temperatures they experience, but this overlooks the potential for local adaptation (Bennett et al., 2015; Vilà‐Cabrera et al., 2019).

Cetaceans are particularly vulnerable to climate change (MacLeod, 2009) due to their crucial function as apex predators in marine ecosystems (Bowen, 1997). Climate change influences the availability of their prey and modifies the habitat, thereby impacting several (if not all) cetacean species and may even put some of them at a high risk of extinction (Learmonth et al., 2006). As a response, several cetacean species are shifting their distribution ranges to track suitable habitats or adapt locally by switching to different food resources (Lambert et al., 2011; Learmonth et al., 2006; MacLeod, 2009; Williamson et al., 2021). For example, temperate and subpolar small cetaceans like the harbor porpoises (*Phocoena phocoena*) are likely to show a poleward shift (Heide‐Jørgensen et al., 2011; MacLeod, 2009). Due to an elevated metabolic rate and relatively short generation time, this small cetacean is heavily dependent on a continuous food supply (Hoekendijk et al., 2018; Wisniewska et al., 2016). This species is thus expected to be particularly sensitive to climate changes (Heide‐Jørgensen et al., 2011). In addition, due to their coastal distribution, thousands of harbor porpoises are also killed accidentally each year by commercial fisheries. This by‐catch mortality is threatening local populations to a level that is still hard to quantify, but it is likely unsustainable, especially in a highly fluctuating environment (Carlén et al., 2021; IMR‐NAMMCO, 2019; Vingada & Eira, 2018).

**FIGURE 1:**
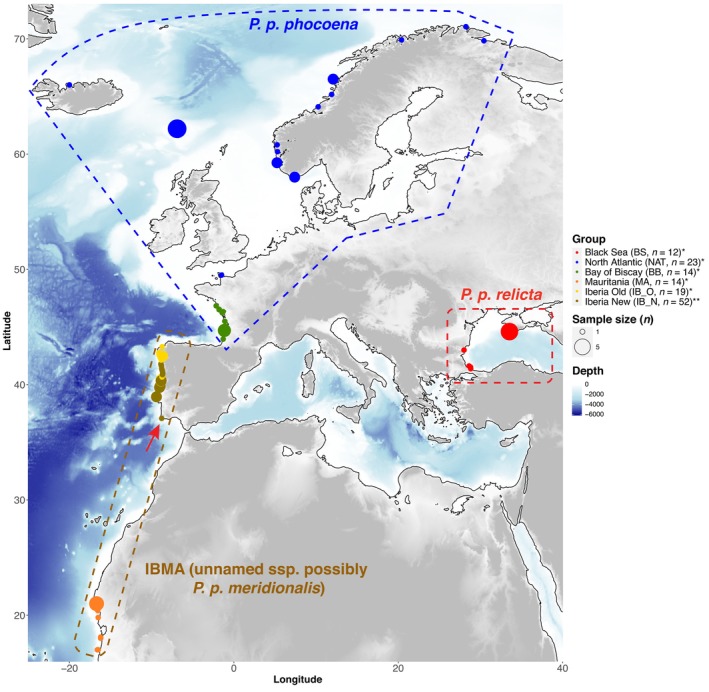
Map showing the sampling locations and sample sizes. Geographic locations are based on approximate GPS coordinates or reported discovery locations. The red arrow indicates the geographical location of the two porpoises carrying Haplotype 10 (*Fontaine et al., 2014; **This study).

Harbor porpoises are officially subdivided into three distinct subspecies: *P. p. vomerina* in the Pacific, *P. p. phocoena* in the North Atlantic, and *P. p. relicta* in the Black Sea (Fontaine, 2016; Read, 1999; Rosel et al., 1995). A fourth lineage or Evolutionary Significant Unit (ESU) has been suggested for the Afro‐Iberian harbor porpoises (IBMA; currently unnamed subspecies, but possibly *P. p. meridionalis* following Fontaine et al., 2014) owing to their distinctive ecology, morphology (Donovan & Bjørge, 1995; Smeenk et al., 1992), and genetic divergence (Fontaine et al., 2014) (Figure 1). However, no formal description or subspecies taxonomic assignment has been made for this distinct lineage so far (Pierce et al., 2020). IBMA porpoises are found around the relatively cold and productive upwelling waters off the coasts of Iberia and on the NW Atlantic coasts of Africa from southern Morocco, to Mauritania, and Senegal (Fontaine, 2016; Gaskin, 1984; IMR‐NAMMCO, 2019; Read, 1999). They are significantly larger in terms of skull morphology, growth curve, and adult body size (typically ≥2 m long) compared with the two other ecotypes (or subspecies) (~1.5 m long) (Galatius & Gol'din, 2011; Read, 1999; Sequeira, 1996; Smeenk et al., 1992). Besides their distinctive morphological features, the IBMA porpoises belong to the Eastern Canary Coastal province with equatorward surface circulation in the form of the Canary current, rich in eddies and large upwellings that are permanent along the NW African coast and seasonal along the Iberian coast (Álvarez‐Salgado et al., 2003; Fraga, 1981; Moustahfid et al., 2021; Pierce et al., in press). While *P. p. phocoena* in the northern Bay of Biscay and further north in the North Atlantic are generally considered opportunistic feeders relying primarily on bottom‐dwelling on the shallow continental shelf (i.e., in waters of <200 m depth), the IBMA porpoise feeding ecology is still poorly known. It is hypothesized that they exhibit similar foraging behaviors as their counterparts on the continental shelf to the north, but with a greater emphasis on pelagic fish (Pierce et al., in press; Pinela et al., 2010; Santos & Pierce, 2003). From a genetic perspective, the Iberian and Mauritanian porpoises are closely related but they were found genetically differentiated from each other and form two distinct populations separated by at least 2500 km (Ben Chehida et al., 2021; Fontaine, 2016; Fontaine et al., 2014).

The upwelling habitat of the Iberian porpoise (IB) population is at the border of a biogeographical transition zone between temperate and subtropical waters where species with different affinities co‐occur and reach their respective ecological limits (Bowen, 1997; MacLeod, 2009). Because species' habitat suitability at the margins between biogeographic zones is often suboptimal, populations like the Iberian harbor porpoises are expected to be among the first to show the impacts of environmental changes. This is particularly true with the small‐scale Iberian upwelling system, which is known to fluctuate in response to ongoing climate change (Casabella et al., 2014; Pires et al., 2013). Such environmental fluctuation may impact the Iberian harbor porpoises in various ways, possibly leading to dramatic impacts on the population demography and genetic diversity. Porpoises may adapt locally, for example, by adapting to various prey species (Heide‐Jørgensen et al., 2011), by migrating to more suitable environments, or the population may also become extirpated if environmental changes outpace their ability to adapt. Previous population genetic studies (Ben Chehida et al., 2021; Fontaine et al., 2007, 2010, 2014, 2017), based on samples collected between 1990 and 2002, showed that the Iberian population was a source population with highly asymmetric gene flow, sending more migrants to the North into the *P. p. phocoena* populations on the northern side of the Bay of Biscay and to the South into the Mauritanian population than in the other direction (Figure 1). In fact, a tension zone was detected on the northern side of the Bay of Biscay with Iberian genetic ancestry decreasing sharply with the increasing latitude (Fontaine et al., 2007, 2014, 2017). If recruitment does not keep up with the mortality and migration rate, the census size of the Iberian population may decrease, leading to a gradual reduction in number of reproducing individuals, in genetic diversity, and thus in effective population size over time.

Unlike previous studies, which inferred population structure between the Iberian population and other populations (Ben Chehida et al., 2021; Fontaine et al., 2007, 2010, 2014, 2017), the goal of the present study is to assess how mtDNA variation evolved over time in the Iberian population, comparing sampling cohorts spanning about three decades and how this information can guide conservation of the Iberian population. More precisely, using genetic diversity as a proxy for effective population size variation, we tested if mitochondrial genetic diversity decreased through time by comparing the genetic composition of two cohorts of porpoises, one sampled in the 1990s (1990–2002), and the other one from the 2010s (2012–2015).

## 2 MATERIALS AND METHODS

### 2.1 Sampling and molecular analyses

Tissue samples from 52 by‐caught or stranded individuals collected along the Portuguese coast between 2012 and 2015 were obtained from the Portuguese Marine Animal Tissue Bank (MATB). These samples form the 2010s cohort of the Iberian population (also called the “new” cohort or IB_N in this study) (Figure 1, Table 1, and Table S1). Tissue samples were stored in 70% ethanol and kept at −20°C until molecular analyses. Genomic DNA was extracted with the DNeasy Blood and Tissue kit (QIAGEN Inc.) following the manufacturer's protocol. Following Fontaine et al. (2014), we screened five mitochondrial coding regions (*ATP‐6*, *ATP‐8*, *Cyt‐b*, *ND5*, and *COI*) for genetic polymorphisms using Sanger sequencing. Target regions were PCR amplified, quality controlled, and purified following the protocol described in Fontaine et al. (2014). Sanger sequencing was outsourced to GATC Biotech Ltd. The resulting forward and reverse sequences were visually inspected, cleaned, and assembled with *Geneious* 10.0.9 (Kearse et al., 2012).

**TABLE 1:** Mitochondrial genetic diversity.

	*N*	*S*	*Singl*.	*Parsim*.	*# Hap*	*Hd*	*π (%)*	*θ*_*W*_ (%)	*D*	*Y*
BS^1^	12	23	21	2	10	0.96	0.10	0.18	−2.04	−1.56
NAT^1^	23	99	57	42	18	0.97	0.41	0.64	−1.46	−0.83
BB^1^	14	66	19	47	10	0.95	0.52	0.50	0.22	0.05
MA^1^	14	11	1	10	7	0.90	0.07	0.08	−0.66	−1.93
All IB^2^	71	27	7	20	17	0.72	0.05	0.13	−1.85	−2.12
IB_O^1^	19	12	6	6	10	0.74	0.05	0.08	−1.53	−1.77
IB_N^3^	52	24	5	19	11	0.70	0.06	0.13	−1.77	−2.19
IB_N^a^ ^3^	50	14	7	7	10	0.73	0.04	0.08	−1.58	−1.26
All	134	170	65	105	60	0.91	0.40	0.74	–	–

*Note*: The statistics display per group and overall include the mtDNA sample sizes (*N*), the number of segregating sites (*S*), singleton mutations (*Singl*.); sites informative in parsimony (*Parsim*.), number of haplotypes (*# Hap)*, haplotype diversity (*Hd*), the nucleotide diversity (*π*), Watterson's theta (*θ*_*W*_), Tajima's *D*, *Y*, Achaz's *Y*. Population codes are as follow: BS, Black Sea; NAT, North Atlantic; BB, Bay of Biscay; MA, Mauritania; IB_O, Iberian Old 1990s cohort; IB_N, Iberian new 2010s cohort. Superscript next to each population code indicates the origin of the mtDNA sequence: ^1^Fontaine et al. (2014), ^2^Fontaine et al. (2014) + this study, ^3^This study. See Table S1 for more details on the samples.

^a^ Excluding haplotype 10.

We augmented the data set with the 82 previously published sequences from Fontaine et al. (2014) encompassing the same five mitochondrial genes. These sequences were obtained for 82 harbor porpoises collected between 1990 and 2002 (Figure 1, Table 1 and Table S1). These included: 14 individuals collected along the French coast of the Bay of Biscay (BB), 23 further north in the North Atlantic (NAT) from the Channel to the Nordic waters of Norway and Iceland; 19 from the Iberian coast (*a.k.a*. the “old” Iberian cohort or IB_O), 14 from Mauritania (MA); and 12 from the Black Sea (BS). The old Iberian cohort from Fontaine et al. (2014) obtained between 1990 and 2002 included 19 stranded and bycaught porpoises from the Galician coasts of Spain and Portugal (Figure 1 and Table S1). Previous studies based on mtDNA data and nuclear microsatellite data demonstrated that the Spanish and Portuguese samples were part of the same panmictic genetic group (Ben Chehida et al., 2021; Fontaine et al., 2007, 2014, 2017). The main focus of the present study thus consists in comparing the temporal evolution of the mtDNA variation between the two Iberian cohorts of porpoises: that is, the new (2010s: 2012–2015, *n* = 52) versus the old (1990s: 1990–2002, *n* = 19). The temporal coverage of this serial sampling of the Iberian population is thus at most 25 years, which corresponds to 2.5 generations, assuming a generation time of 10 years (Read, 1999). The mtDNA sequences from the other populations of Fontaine et al. (2014) neighboring the Iberian population were included here to provide a broader perspective to interpret the phylogenetic analyses.

One additional sequence from the closest outgroup species, the Dall's porpoise (*Phocoenoides dalli*) also generated by Fontaine et al. (2014), was included in the present data set to root the mitochondrial phylogeny. All mtDNA sequences (*n* = 135) were aligned with *MUSCLE* (Edgar, 2004).

### 2.2 Phylogenetic and population genetic analyses

In order to place the Iberian porpoises into a global phylogeographic context, we first reconstructed a maximum‐likelihood (ML) phylogeny with all the 135 mtDNA sequences (including the outgroup). Unique mtDNA haplotypes were identified using *DnaSP* v.5.10.1 (Librado & Rozas, 2009). Then, we used *PhyML* (Guindon et al., 2010) implemented in *Geneious* to estimate the ML tree based on all available unique haplotypes identified in the dataset using a nearest neighbor interchange topology search. We used the Tamura and Nei (1993)'s (or TN93) model of nucleotide evolution as identified as the best‐fitting model for our sequence alignment using the Akaike Information Criterion (AIC) in jModelTest 2.1.10 (Darriba et al., 2012). Each node's support was evaluated by conducting 5000 iterations of bootstrap resampling. The phylogenetic tree was rooted with the sequence from a Dall's porpoise. In addition to the phylogenetic trees, we also reconstructed a median‐joining haplotype network (Bandelt et al., 1999) using *PopART* (http://popart.otago.ac.nz).

Various statistics informative on the mtDNA genetic variation of the two cohorts of Iberian porpoises (old vs. new) were estimated using the C library *libdiversity* (https://bioinfo.mnhn.fr/abi/people/achaz/cgi‐bin/neutralitytst.c) and the R package *pegas* v.1.2 (Paradis, 2010) (Table 1). Genetic diversity was estimated using the number of haplotypes (*H*), haplotype diversity (Hd), nucleotide diversity (*π*), and Watterson's theta (*θ*_*W*_). Differences in sample size can significantly impact the values of some genetic diversity estimators (Goodall‐Copestake et al., 2012). Therefore, a rarefaction approach was applied to estimate measures of genetic diversity while correcting for differences in sample size (Sanders, 1968) using a custom Python script. More precisely, for the two cohorts of Iberian porpoises, for each statistic and each sample size increment *k*, we randomly subsampled 5000 times with replacement a number of *k* sequences available in each group, with *k* ranging between 2 and the *k*_max_ available sequences (up to a maximum of 50). At each sample size increment *k*, we estimated the mean and standard error values per statistic and per group. Subsequently, genetic differentiation between the two cohorts was assessed using Hudson's estimator of *F*_ST_ (Hudson et al., 1992) and *φ*_ST_ (Excoffier et al., 1992) using, respectively, *the libdiversity C‐library* and the R‐package *haplotypes v.1.1.3.1*. The significance levels of *F*_ST_ and *φ*_ST_ were assessed by randomization tests (1000 permutations). Finally, we investigated evidence of changes in mtDNA effective population size (*Ne*) between the two cohorts (old vs. new) by calculating values of Tajima's *D* (Tajima, 1989) and Achaz's *Y* (Achaz, 2008) accounting for differences in sample sizes by implementing the same rarefaction approach as described for the diversity statistics. Achaz's *Y* statistic is analogous to Tajima's *D*, but accounts for potential sequencing errors (see details in Achaz, 2008).

### 2.3 Approximate Bayesian computation (ABC)

**FIGURE 2:**
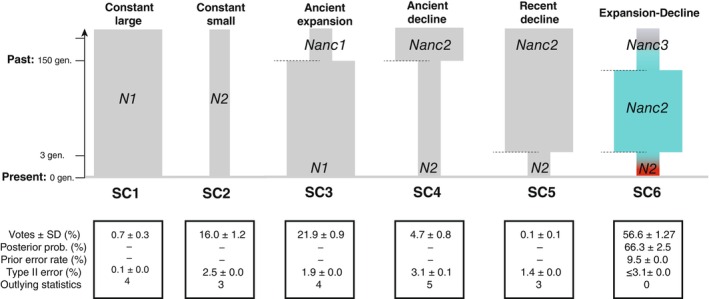
Alternative scenarios were tested in the ABC‐RF procedure. See Table S2 for further details on the model parameters. Below each scenario are provided the main results including the number of Random Forest (RF) votes (in percent), the posterior probability of the best scenario, the prior error rate, the type II error rate, and the number of outlying statistics in the goodness‐of‐fit testing (see Table S5). The colored scenario, SC6, was the scenario gathering the highest number of Random Forest votes. The time scale is indicated by the arrow on the left, in generations before present (gen.).

In order to evaluate the support for different demographic scenarios and assess whether there is a signal of a recent population decline in the Iberian population based on the mtDNA data, we applied an ABC approach relying on coalescent simulations (Lopes & Beaumont, 2010). More precisely, we compared six demographic scenarios: (1) a population with a large constant effective population size, (2) a small constant effective population size, (3) an old expansion (≥150 generations, gen.), (4) an old decline (≥150 gen.), (5) a recent decline (≤3 gen.), and (6) an old expansion (>150 gen.) followed by a recent decline scenario (≤3 gen.) (see Figure 2 and Table S2 for details). In the coalescent simulations, we leverage the time series data information available and specify explicitly at which time the samplings of the two cohorts were performed.

For each of the six scenarios, we ran 10,000 coalescent simulations using *fastsimcoal2 v2.6* (Excoffier & Foll, 2011). Details on the coalescent simulations and the mutation model used are provided in Table S2. We used the program *Arlsumstat v3.5*.2 (Excoffier & Lischer, 2010) to compute a set of 55 summary statistics listed in Table S3, augmented with the principal axes obtained from a linear discriminant analysis (LDA) on the summary statistics (Pudlo et al., 2016), to describe and summarize the genetic variation of each simulation.

We employed a random forest classification (RF) procedure for the model selection (Pudlo et al., 2016). RF is a machine‐learning algorithm that uses bootstrapped decision trees to classify and select the best scenario using the summary statistics as a set of predictor variables (Breiman, 2001). RF was therefore applied to compare the likelihood of the six competing scenarios (Figure 2). This procedure provides classification votes that account for the number of times a scenario is selected as the best one among the trees used to construct the random forest. Based on a forest of 1000 decision trees, the scenario that gathered the highest number of votes was selected as the most likely.

The *prior* error rate and the type II error rate were estimated using the out‐of‐bag simulations (i.e., simulations not used to build the decision trees). In addition, we computed the confusion matrix, which provides a more global assessment of the performance of our RF procedure (Schrider & Kern, 2018). The convergence of the results was evaluated over 10 independent RF runs, as recommended by Fraimout et al. (2017).

Finally, to check whether the formulated scenarios and associated priors were compatible with the observed data set, we plotted both the simulated data sets from the training set and the observed data set on the two first principal axes of a linear discriminant analysis (LDA) and of a principal component analysis (PCA), following the recommendation of Pudlo et al. (2016). We visually checked whether the summary statistics from the observed data were located within the clouds formed by those from the simulated data for at least one demographic scenario. In addition, we assessed the goodness‐of‐fit between the simulated and the observed data following Gelman et al. (2004): the summary statistics for which the cumulative simulated distribution was larger or smaller than 95% CI of the distribution compared to the observed value were considered as significantly different from the observed data. All ABC‐RF analyses were performed using the R package *abcrf* v.1.8.1 (Pudlo et al., 2016) in R 4.1.2 (R Core Team, 2016).

## 3 RESULTS AND DISCUSSION

In total, we successfully sequenced 52 Iberian harbor porpoises from the new cohort (2012–2015) for fragments of five mitochondrial genes including Cytochrome b (Cyt‐b: 1111 base pairs, bp), and ATP‐6 and ‐8 (842 bp), NADH dehydrogenase 5 (ND5: 707 bp), and the Cytochrome c oxidase subunit I (COI: 1515 bp), When aligned with the 82 previous sequences of harbor porpoises from Fontaine et al. (2014) and an additional one of the Dall's porpoise used here as an outgroup, the final alignment of 4175 base pairs (bp) included 135 sequences and contained 384 segregating sites with 267 singletons and 117 parsimony informative sites defining 61 distinct haplotypes. Without the outgroup, the 134 mtDNA sequences of harbor porpoise contained 170 segregating sites with 65 singletons and 105 parsimony informative sites defining 60 distinct haplotypes (Table 1).

**FIGURE 3:**
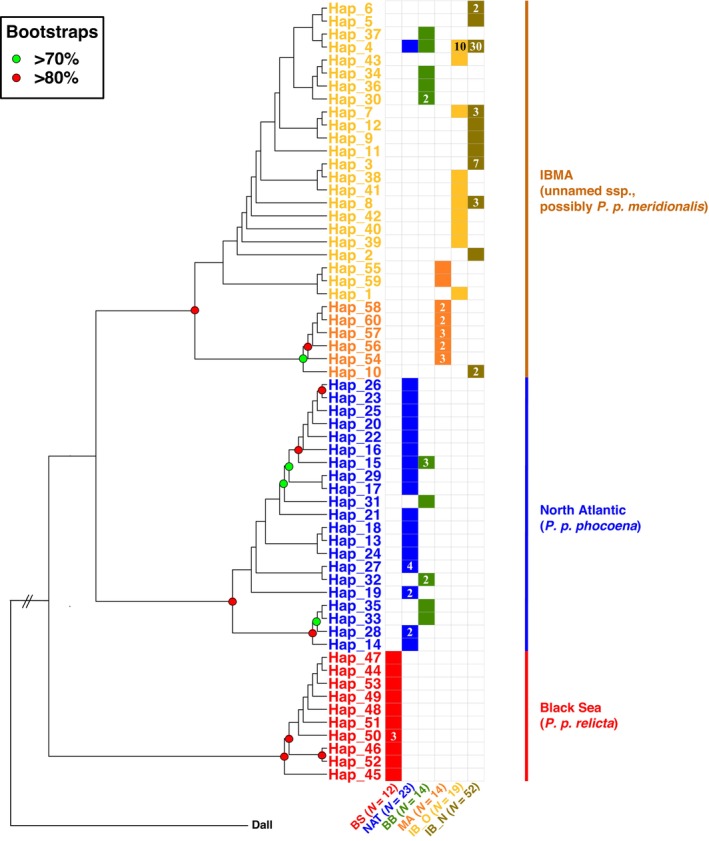
Cladogram derived from the maximum‐likelihood mitochondrial phylogeny among unique mitochondrial haplotypes. A branch length transformation proportional to the number of tips under the node (as implemented in *FigTree*) was applied to facilitate the visualization of the branching patterns (see Figure S1 for the actual phylogenetic tree with branch lengths proportional to sequence divergence). The color‐coded labels show the geographic origin of the haplotype. The numbers within the boxes refer to the number of individuals carrying the haplotype. No number in a box means that the haplotype was observed only once. Population code are as follow: BS, Black Sea (*N* = 12)*; NAT, North Atlantic (*N* = 23)*; BB, Bay of Biscay (*N* = 14)*; MA, Mauritania (*N* = 14)*; IB_O, Iberian Old (*N* = 19)*; IB_N, Iberian new (*N* = 52)** (*Fontaine et al., 2014; **This study).

Consistent with previous studies, the phylogenetic relationships among mtDNA haplotypes depicted by the ML tree (Figure 3 and Figure S1) and by the haplotype network (Figure S2) revealed the three main monophyletic lineages corresponding to the three previously identified subspecies or ESU's (Fontaine et al., 2014): (1) *P. p. relicta* in the Black Sea, (2) *P. p. phocoena* composed of the porpoises from the North Sea to the waters of Norway, and (3) the Afro‐Iberian (IBMA) porpoises (unnamed *ssp*. possibly *P. p. meridionalis*), which comprises two distinct monophyletic sub‐lineages in the upwelling waters of Iberia and Mauritania, respectively.

Evidence of migration from the Iberian to the Mauritanian populations has been previously documented (Ben Chehida et al., 2021; Fontaine et al., 2014) and is also observed here with two haplotypes (Hap_55 and Hap_59) found in the Mauritian population, but clustering with the Iberian lineage (Figure 3, Figures S1 and S2). However, we also identified one haplotype (Hap_10 or Haplotype 10, see Figure 3, Figures S1 and S2) present in two porpoises from the new Iberian cohort, but clustering with the Mauritanian lineage. This haplotype was carried by two individuals in the southernmost part of the Iberian sampling (red arrow in Figure 1). This result provides the first evidence of possible dispersal (or migration) from the Mauritanian population into Iberian waters, suggesting that these two populations may not be as isolated as previously thought. Haplotype 10 could also belong to a distinct unknown population as it is relatively divergent from the other Mauritanian haplotypes (Figure 3, Figures S1 and S2). Indeed, the ML tree (Figure 3 and Figure S1) shows that this haplotype forms a strongly supported (bootstrap support > 70%) distinct lineage from the other Mauritanian porpoises. The haplotype network also illustrates this divergence (Figure S2): while the Mauritanian haplotypes are separated from each other with at most two mutational steps, at least 10 mutational steps separate this haplotype 10 from the other Mauritanian haplotypes (Figure S2). Notably, both porpoises carrying this haplotype 10 were found on the southern extremity of the Portuguese coasts (8.78 W–37.07 N; Praia do Burgau, Portugal), at least 400 km south of all other Iberian sampling locations (Figure 1). Stranding records of harbor porpoises were previously reported in Morocco, on the east coasts of Gibraltar, and in Cadiz (Spain; Rojo‐Nieto et al., 2011). If unsampled populations occur between the Iberian Peninsula and the Mauritanian waters, they are most likely confined to a narrow and seasonal upwelling area. This relatively divergent haplotype 10 closely related to the Mauritanian rather than the Iberian porpoises could be evidence of such unsampled populations. This hypothesis of such an unsampled population could be plausible since harbor porpoise sightings, stranding, and bycatches have been reported from the North of Morocco to the Gambia (Boisseau et al., 2010; IMR‐NAMMCO, 2019). Haplotype 10 was excluded from subsequent analyses of genetic diversity and coalescent simulations because it could be part of a distinct population and therefore would artificially inflate measures of genetic diversity (Table 1 and Table S1). Future studies with additional samples from these relatively unsampled regions (southern Iberian coast and Morocco) and data from the nuclear genome are needed to confirm whether these two porpoises are genetically distinct from the other Iberian porpoises, possibly forming a distinct population, and whether or not they are admixing with the other Iberian porpoises.

**FIGURE 4:**
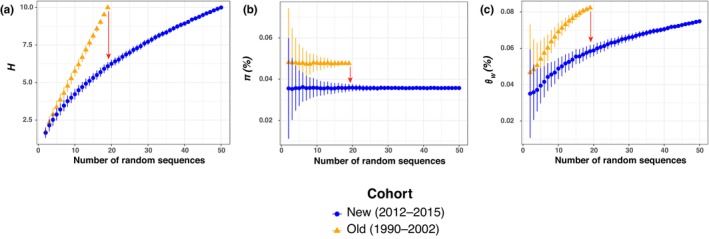
Rarefaction curves showing three statistics describing mitochondrial genetic diversity including (a) the number of haplotypes (*H*), (b) the nucleotide diversity (*π*), and (c) Watterson's theta (*θ*_*W*_). The red arrow in each panel shows the direction of change between the old and new cohorts. The *X*‐axis shows the number of sequences randomly resampled in the rarefaction procedure to estimate the statistical values. The mean and standard error are provided at each sample size increment (*k*) for each statistic and each group, calculated from 5000 sampling with replacement of the *k* sequences available in each group, with *k* ranging from 3 to *k*_max_ up to 50.

Aside from the two porpoises carrying the haplotype 10, our results indicate that both cohorts sampled at different times along the Iberian coasts belong to the same mtDNA gene pool. We detected no genetic differentiation between the two Iberian cohorts suggesting no significant differences in haplotype frequencies between them (*F*_ST_ and *φ*_ST_ values = 0; *p*‐value > .54, Table S4). This result reflects the high prevalence of haplotype 4 which dominates in both cohorts (Figure 3, Figures S1 and S2). However, once we accounted for differences in sample size between the two cohorts using a rarefaction method, we observed a significant reduction in genetic diversity in the new cohort compared with the old one as shown by the lower number of haplotypes (*H*; Figure 4a), and in the two measures of nucleotide diversity (*π* and *θ*_*W*_; Figure 4b,c, respectively). Rarefaction curves provided striking evidence of this reduction in genetic diversity (Figure 4). The nonoverlapping standard errors from a standardized sample size beyond eight sequences suggest that the decline of genetic variation is likely highly significant. For example, the average number of haplotypes (*H*) observed from 19 sampled sequences in each cohort decreases from 10 to 6 when comparing the old versus new cohorts. This suggests that the proportion of rare haplotypes has dropped substantially over the 25 years sampling period of this study. Consistent with this observation is the rapid loss in nucleotide genetic diversity as measured by *π* and *θ*_*W*_ (Figure 4b,c). Such a substantial loss of genetic variation over 2.5 generations is often attributed to an enhanced effect of genetic drift rather than other evolutionary forces like selection (Hartl, 2020). This could suggest a dramatic decline of the Iberian population in terms of effective population size, possibly reflecting a substantial decline in the number of reproducing individuals.

**FIGURE 5:**
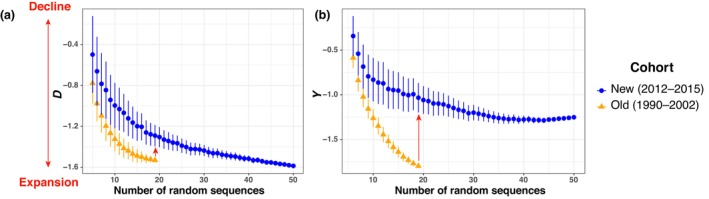
Rarefaction curves showing two neutrality indices indicative of demographic changes including (a) Tajima's *D* and (b) Achaz's *Y*. The red arrow inside each panel shows the direction of the change between the old and new cohorts. The bidirectional arrow shows the interpretation of *D* and *Y* in terms of demography. The *X*‐axis shows the number of sequences randomly resampled in the rarefaction procedure to estimate the statistical values.

Tajima's *D* (Tajima, 1989) and Achaz's *Y* (Achaz, 2008) statistics estimated for the two cohorts sampled at different times along the Iberian coast also displayed negative values (Figure 5). At first glance, such negative values would be indicative of a population that underwent a “recent” expansion at evolutionary time scales (Epps & Keyghobadi, 2015; Smith et al., 2011), most likely due to the foundation of the Iberian population during the period that followed the Last Glacial Maximum (19 kyr ago; Fontaine et al., 2014; Fontaine, 2016). However, the values estimated for the new cohort for both statistics were significantly less negative (based on nonoverlapped standard error estimates) than those from the old cohort. For example, Tajima's *D* values obtained from 19 randomly sampled sequences increased from −1.5 for the old cohort to −1.25 for the new (Figure 5a). The change in Achaz's *Y* values, which account for possible sequencing errors (Achaz, 2008), shows an even stronger increase from −1.75 to −0.95 between the old and new cohorts (Figure 5b). It is remarkable to see how both values have become dramatically less negative in the time lapse of only 2.5 generations between the two cohorts. This increase in *D* and *Y* values also provides a strong indication of a population losing rare haplotypes as it would be expected for a small and isolated population strongly subject to genetic drift that could be declining.

Such a loss of genetic diversity may reflect different demographic scenarios, for example, a small population that did not experience any population size changes, but still experienced strong genetic drift. Alternatively, a scenario of ancient expansion and a very recent decline could be also possible. We used a simulation‐based Approximate Bayesian computation analysis relying on the random‐forest classifier (ABC‐RF) to explore statistically six plausible demographic scenarios (Figure 2) describing the evolutionary history of the Iberian population since it diverged from the Mauritanian population within the last 150 generations (Fontaine et al., 2014). Out of the six scenarios compared, the analysis strongly supported the SC6 scenario assuming an old expansion (>150 generations) followed by a recent decline with the past three generations (SC6; Figure 2, Figures S3–S5). Ten replicates of the ABC‐RF analysis supported strongly and unanimously this result (Figure S3). Scenario SC6 gathered in average (±SD) over the 10 runs 56.6 ± 1.3% of the RF votes (Figure 2 and Figure S3), with a posterior probability of 66.3 ± 2.5%, a prior error rate of 9.5 ± 0.0%, and type II errors ≤3.1% ± 0.0%. Furthermore, the confusion matrix (Figure S5) depicting the overall performance of the RF classification analysis indicated high power to distinguish between the competing scenarios (>90%). Such a high‐resolution power to discriminate among the different demographic scenarios likely comes from the multiple sampling time points included in the ABC analysis. The goodness‐of‐fit analysis provided additional support to the demographic model identified by showing that scenario SC6 was the only scenario that produced simulated values that closely matched the observed values for each summary statistic used in this analysis (Table S5, Figures S4 and S6). This scenario SC6 (Figure 2) suggests that the effective size (*Ne*) of the Iberian population expanded at least ~150 generations ago, thus ~1500 years ago, assuming a rough generation time of 10 years (Fontaine et al., 2014). Then, under this scenario, *Ne* values of the Iberian population would have declined by a factor ≥ 10 within the last three generations (thus 30 years). Thus, this ABC analysis, together with the rarefaction results, adds an additional line of evidence suggesting a rapid loss of genetic variation over the last three generations due to a very recent decline in effective population size. The reader should note, however, that the effective population size (*Ne*) is a population genetic concept that differs from and is usually smaller than the *census* population size. It does not represent the number of individuals actually alive at any given time point. Following Frankham (2019), *Ne* is the number of individuals that would result in the same loss of genetic diversity, inbreeding, genetic drift, etc., if they behaved in the manner of an idealized population. Such an idealized population would assume nonoverlapping generations, random mating, and no migration, mutation, or selection. *Ne* would thus roughly represent the number of breeding individuals in this idealized population, or more precisely the number of breeding females since we analyzed here maternally inherited fragments of the mitogenome that behave as a single nonrecombining haploid unit. Therefore, our results are not intended to estimate exactly how many Iberian porpoises there were at different points in history, but rather they suggest that the number of reproducing females may have been substantially declining in the Iberian population over the last 2.5 generations. However, such interpretation must be taken with caution considering all the assumptions related to the *Ne* concept.

In this study, we also confirmed previous evidence of gene flow from the Iberian population to more northern populations with Iberian haplotypes present in the Bay of Biscay and in the English Channel where admixture between the two lineages, IBMA (or *P. p. meridionalis*) and *P. p. phocoena*, has been reported (see Figure 3, Figures S1 and S2; Table S4, and also Ben Chehida et al., 2021; Fontaine et al., 2014, 2017). Additionally, we did not find any evidence of gene flow from the populations located further North into the Iberian population, as previously reported (Ben Chehida et al., 2021; Fontaine et al., 2007, 2010, 2014, 2017). Indeed, Fontaine et al. (2014) estimated very asymmetric values of gene flow using the coalescent approach of MIGRATE (Beerli, 2006, 2009) based on microsatellite data. While no evidence of immigration was detected toward the Iberian porpoises from the neighboring populations, ca. 3 individuals per generation were estimated migrating from the Iberian to the Mauritanian populations, and 70 per generation from the Iberian to the northern Bay of Biscay populations (see tab 2 in Fontaine et al., 2014). In the northern Bay of Biscay and adjacent waters, a geographically restricted admixture zone was reported forming a well‐delimited tension zone (Fontaine et al., 2017). Because the population size of *P. p. phocoena* in the North Sea and adjacent waters is much larger than that of the Iberian population (census *N* estimates from the SCANS‐III survey (Hammond et al., 2017): ~400,000 vs. ≤3000 individuals respectively), the observed northward migration may possibly lead to the assimilation of the Iberian gene pool by *P. p. phocoena*. In the long term, this process could be an additional cause of concern as it could result in the disappearance of the unique Iberian genetic pool. This scenario is advanced by MacLeod (2009), which used habitat modeling to show that species with a similar range as the Iberian harbor porpoises (fig. 1c in MacLeod, 2009) may shift their range northward in response to a 5°C increase in water temperature due to global warming (fig. 1d in MacLeod, 2009). This model suggests that the future range of IBMA could completely overlap with the current range of *P. p. phocoena* (fig. 1d in MacLeod, 2009). It is worth mentioning that Ben Chehida et al. (2021) recently modeled the future distribution of the harbor porpoises in the North Atlantic under the most aggressive scenario of the Intergovernmental Panel on Climate Change (IPCC) in 2050 (RCP8.5; Schwalm et al., 2020) and did not capture this strong northward range shift. The discrepancy between the two models may be related to differences in assumed temperature increase, 2 and 5°C, in Ben Chehida et al. (2021) and MacLeod (2009), respectively. In addition, the environmental niche modeling in Ben Chehida et al. (2021) did not account for any biotic factors in the simulations. However, environmental niche modeling such as those conducted by MacLeod (2009) and Ben Chehida et al. (2021) needs to be taken with some caution since they do not account for local population adaptation. In the case of IBMA porpoises, their morphological and genetic distinctiveness together with the peculiar upwelling habitat they inhabit suggest that they are adapted to the local conditions. Future genomic studies could assess whether selective processes have impacted the genome of IBMA porpoises and whether there is genetic evidence of such a local adaptation process compared with other populations.

The results of this study support the hypothesis that the Iberian population is both small and relatively isolated. Furthermore, both the sharp decline in mtDNA genetic diversity over the past 25 years and northward migration may represent reasons for concern for the long‐term persistence of the Iberian porpoises. One plausible explanation is that the decay of mtDNA genetic variation we reported here could simply reflect the fact that Iberian porpoises constitute a stable population with a very low effective population size (*Ne*) (SC2 in Figure 2). This scenario SC2 was not the best model according to our simulation‐based ABC‐RF analysis, but the third best one, receiving only 16% of the total RF classification votes, but it remains plausible (Figure 2, Figures S3–S6 and Table S5). In such a condition, genetic drift would have a disproportionate influence on the genetic makeup of this population and could lead to the observed loss of genetic variation over a few generations. Several previous studies showed that the Iberian population has indeed a very low *Ne* at nuclear microsatellite loci (Ne < 100 individuals; Ben Chehida et al., 2021; Fontaine et al., 2010, 2014). The mtDNA employed in this study has an effective size four times smaller compared with nuclear autosomal loci. This means that the stochastic loss of genetic variation due to genetic drift would be drastically increased for mtDNA compared with nuclear loci. Thus, the Iberian population might be at a stable census size losing rare haplotypes through the enhanced effect of genetic drift at the mtDNA. Another more plausible explanation receiving the highest support in our ABC‐RF analysis (scenario SC6, Figure 2, Figures S2–S6, Table S5) is that the decay of mtDNA genetic variation might be associated with a rapid and recent decline in the number of reproductive females in addition to the overall low *Ne* (Lande, 1988), as depicted by the scenarios SC6 in Figure 2. This scenario SC6 supports a severe population decline in the last three generations, leaving at most 10% of the population predecline *Ne*. This hypothesis is even more plausible since there is accumulating evidence that the Iberian population is sending more migrants to adjacent populations than the reverse (Ben Chehida et al., 2021; Fontaine et al., 2014, 2010). Thus, in addition to being small and isolated, the recent and strong population decline envisioned under SC6 of the ABC‐RF analysis would imply that the Iberian porpoises may become increasingly subject to demographic stochasticity (Allendorf et al., 2012). Because this population is also known to be subject to strong genetic drift and environmental stochasticity (Casabella et al., 2014; Pires et al., 2013), all these effects can accumulate and reinforce each other, potentially precipitating the decline of the Iberian porpoises. This so‐called extinction vortex (Allendorf et al., 2012) can be particularly severe for a long‐lived *K* strategist species, inhabiting a marginal habitat highly subject to environmental variation like the Iberian harbor porpoises (Casabella et al., 2014; Pires et al., 2013) and could put them at a high risk of extinction.

The present study relies on a single molecular marker (mtDNA). Therefore, future studies with additional samples and data from the nuclear genome are highly needed to confirm the trends highlighted here. Indeed, genomewide data can provide further insights into the temporal dynamics of the Iberian population. For example, the reconstruction of historical demography based on genomewide data (Liu & Fu, 2020; Schiffels & Wang, 2020; Terhorst et al., 2017) can be used to test whether these harbor porpoises maintained a long‐term low *Ne* (Morin et al., 2020; Robinson et al., 2022) since their postglacial emergence (Fontaine, 2016; Fontaine et al., 2010, 2014), or if on the contrary, the population declined recently (Hu et al., 2020). A simulation framework, similar to the one used by Robinson et al. (2022), could be applied to assess whether these porpoises are already facing deleterious effects of the mutation load, despite their current low *Ne*, or if purging processes have been ongoing similar to those recently reported for the vaquita (*Phocoena sinus*) (Morin et al., 2020; Robinson et al., 2022).

Additional data sources also point to large fluctuations in population size in the Iberian porpoises and require careful consideration: the SCANS‐III survey in the summer 2016 estimated a census size as low as 2898 animals (CV = 0.32) in Iberian waters and the Bay of Biscay (Hammond et al., 2017). Annual aerial surveys in Portuguese continental waters between 2011 and 2015 estimated an overall average abundance of 2254 porpoises and a corresponding density of 0.09 ind/km^2^ (CV = 21.99%) with large interannual fluctuations. The highest annual abundance estimated for that period was 3207 individuals (CV = 38.14%) in 2013, followed by a sharp decrease in 2014, when only 1653 individuals (CV = 43.27%) were estimated (Torres‐Pereira et al., 2022). López et al. (2013) estimated an abundance of 683 (CV = 0.63, 95%CI: 345–951) animals for the Spanish part of the IBMA range (Galicia and Bay of Biscay). Globally, these low census sizes were associated with particularly high bycatches rate (always ≥8%) with the best estimates varying from 90 to 197 deaths per year (Pierce et al., 2020) to 130–330 deaths per year (Read et al., 2020). However, it is important to mention that these estimates are mainly based on “on‐board observer” and aerial survey data that deliver wide confidence limits. This is due to low observer coverage of fishing activities in the region, low density of porpoises, and estimates from various sources (observer data, strandings, aerial surveys, and interviews). Furthermore, Iberian harbor porpoises are regularly found stranded, often with signs of bycatch mortality, potentially indicating unsustainable mortality rates (IMR‐NAMMCO, 2019; López et al., 2002; Pierce et al., 2020; Read et al., 2020; Torres‐Pereira et al., 2022; Torres‐Pereira, Araújo, et al., 2023; Vingada & Eira, 2018). In fact, based on Portuguese stranding data, an average of 9.19% (CI = 5.25%–16.10%) of the porpoise population was estimated to be removed annually by fisheries between 2011 and 2015, corresponding to an average of 207 porpoises removed per year, which largely surpasses the Potential Biological Removal rate conservatively estimated for the same period (22 porpoises, CI: 12–43) (Torres‐Pereira, Araújo, et al., 2023). An additional cause for concern is related to the potential overexploitation of the feeding resources (Méndez‐Fernandez et al., 2013; Santos & Pierce, 2003). A scarcity in prey availability can be harmful because it enhances interspecific competition, as already reported between harbor porpoises and other marine mammals, for example, with the bottlenose dolphin *Tursiops truncatus* whose ecological niche largely overlaps with the harbor porpoise in some portions of Iberian waters (Méndez‐Fernandez et al., 2013; Spitz et al., 2006). Hostile interactions between the two species have been documented in Iberian waters and hypothesized to cause harbor porpoises to disperse to less productive narrow shelf areas (Alonso et al., 2000; Spitz et al., 2006).

## 4 CONCLUSIONS

The Afro‐Iberian (IBMA) lineage of harbor porpoises was previously recognized as a unique Evolutionarily Significant Unit (Crandall et al., 2000; Moritz, 2002) distinct from the other subspecies described in the N. Atlantic and in the Black Sea. IBMA displays unique features in terms of genetics, morphology, ecology, and evolutionary history following Fontaine et al. (2014) (see also the review of Fontaine, 2016). Nevertheless, they are currently not recognized as a distinct subspecies (Pierce et al., 2020, 2022). There is also currently no mention of these porpoises in the IUCN Red List of Threatened Species despite the multiple threats faced by this vulnerable lineage. Given the results of this study, the previous findings on their genetic distinctiveness from other populations at both mtDNA and nuclear markers (Ben Chehida et al., 2021; Fontaine, 2016; Fontaine et al., 2007, 2010, 2014, 2017), and the previous concerns raised about their viability (IMR‐NAMMCO, 2019; Pierce et al., 2020, 2022), we recommend immediate actions and further research from the community in the following areas: (1) a formal taxonomic description is needed for these IBMA porpoises to be officially recognized as a distinct subspecies, following the guidelines of the *International Commission on Zoological Nomenclature* as recommended by the Taxonomic Committee of the Society for Marine Mammalogy and (2) a better assessment for the Iberian porpoises in term of their life history traits, ecology, behavior, genetics and genomics, and the threats to the viability for this unique group. This knowledge will help design better conservation measures and recommendations. It is indeed paramount to ensure that all measures are taken to gain additional knowledge and to apply effective conservation efforts to protect the Iberian population, as recently underlined by the OSPAR Marine Mammal Expert Group (OMMEG) (ICES‐WGMM, 2021; Pierce et al., 2022). Recently, both countries (Portugal and Spain) updated the conservation status of the harbor porpoises inhabiting Iberian waters: from “Vulnerable” to “Endangered” in Spain (Boletin oficial del estado, 2020) and to “Critically Endangered” in Portugal (Torres‐Pereira, Ferreira, et al., 2023). In Portugal, a new Marine Protected Area (PTCON0063) in the northwestern coast was recently designated, whereas another one was enlarged in the southwestern coast (PTCON00012) in order to incur higher protection for harbor porpoise hotspots in the country (Vingada & Eira, 2018). Nonetheless, none of the conservation and mitigation measures within the areas' management plan (Portaria 201/2019, n.d.) is currently in place and bycatch mortality remains effectively unaddressed. In the future, improving our understanding of the demographic trends and the threats these porpoises are facing is required to devise suitable and tailored larger‐scale conservation plans for the whole Iberian population. Such measures are needed to mitigate the decline of this unique lineage potentially on the brink of extinction.

## AUTHOR CONTRIBUTIONS

**Yacine Ben Chehida:** Formal analysis (lead); investigation (lead); methodology (equal); software (lead); validation (equal); visualization (lead); writing – original draft (lead); writing – review and editing (equal). **Tjibbe Stelwagen:** Data curation (supporting); formal analysis (supporting); investigation (supporting); methodology (supporting); resources (supporting); visualization (supporting); writing – original draft (supporting). **Jeroen P. A. Hoekendijk:** Data curation (supporting); investigation (supporting); methodology (supporting); resources (supporting); writing – original draft (supporting). **Marisa Ferreira:** Resources (equal); writing – original draft (supporting). **Catarina Eira:** Resources (equal); writing – original draft (supporting). **Andreia Torres‐Pereira:** Resources (equal); writing – original draft (supporting). **Lidia Nicolau:** Resources (supporting); writing – original draft (supporting). **Julie Thumloup:** Data curation (equal); investigation (equal); methodology (equal); resources (equal); validation (equal); writing – original draft (equal). **Michael C. Fontaine:** Conceptualization (lead); data curation (lead); funding acquisition (lead); investigation (lead); methodology (equal); project administration (lead); resources (equal); supervision (lead); validation (lead); writing – original draft (lead); writing – review and editing (lead).

## CONFLICT OF INTEREST STATEMENT

The authors declare that they have no conflict of interest.

### OPEN RESEARCH BADGES

This article has earned Open Data and Open Materials badges. Data and materials are available at https://dataverse.ird.fr/dataverse/porpoise_genomics and https://doi.org/10.23708/GOPRGV.

## Supporting information


Data S1:

## Data Availability

The data sets and scripts supporting this study are available in the IRD Porpoises Genetics and Genomics Dataverse Repository (https://dataverse.ird.fr/dataverse/porpoise_genomics) at https://doi.org/10.23708/GOPRGV. Mitochondrial haplotypes were also deposited on the NCBI–GenBank under the Accession Numbers (Accession numbers are listed in Table S1).
