# Supplementary material for: Harbor porpoise losing its edge: Genetic time series suggests a rapid population decline in Iberian waters over the last 30 years
Source: Ecol Evol. 2023 Dec 11;13(12):e10819. doi: 10.1002/ece3.10819 (PMC10714065; doi:10.1002/ece3.10819)
Supplement: Supplementary file 1 — Data S1: [file ECE3-13-e10819-s001.pdf]

ELECTRONIC SUPPLEMENTARY MATERIAL

Harbor porpoise losing its edge: genetic time series suggests a rapid population decline in Iberian waters over the last 30 years

Yacine Ben Chehida[1,2,3], Tjibbe Stelwagen[1,4], Jeroen P. A. Hoekendijk[1,5,6], Marisa Ferreira[7], Catarina Eira[7,8,9], Andreia Torres-Pereira[7,8,9], Lidia Nicolau[7], Julie Thumloup[1], Michael C. Fontaine[1,10]*

Affiliations

[1] Groningen Institute for Evolutionary Life Sciences (GELIFES), University of Groningen, Groningen, The Netherlands

[2] Department of Biology, University of York, Heslington, United Kingdom

[3] Ecology and Evolutionary Biology, School of Biosciences, University of Sheffield, Western Bank, Sheffield, S10 2TN, United Kingdom

[4] BirdEyes, Centre for Global Ecological Change at the Faculties of Science & Engineering and Campus Fryslân, University of Groningen, Zaailand 110, 8911 BN Leeuwarden, The Netherlands

[5] NIOZ Royal Netherlands Institute for Sea Research, Department of Coastal Systems, Utrecht University, Texel, The Netherlands

[6] Wageningen University & Research Centre, Wageningen, The Netherlands

[7] MATB-Portuguese Wildlife Society (SPVS), Figueira da Foz, Portugal

[8] ECOMARE, Universidade de Aveiro, 3810-193 Aveiro, Portugal

[9] Centre for Environmental and Marine Studies CESAM, University of Aveiro, Portugal

[10] MIVEGEC, Univ. Montpellier, CNRS, IRD, Montpellier, France

* **Correspondence to** Michael C. Fontaine (michael.fontaine@ird.fr)
MIVEGEC (U. Montpellier, CNRS, IRD)
Institut de Recherche pour le Développement (IRD),
911 Avenue Agropolis, BP 64501, 34394 Montpellier Cedex 5,
France.

ORCID ID

Yacine Ben Chehida: https://orcid.org/0000-0001-7269-9082
Tjibbe Stelwagen: https://orcid.org/0000-0003-0296-1270
Jeroen P. A. Hoekendijk: https://orcid.org/0000-0002-9308-7429
Marisa Ferreira: https://orcid.org/0000-0002-0733-3452
Andreia Torres-Pereira: https://orcid.org/0000-0002-7189-0771
Catarina Eira: https://orcid.org/0000-0003-2735-6034
Lidia Nicolau: https://orcid.org/0000-0001-5714-162X
Michael C. Fontaine: https://orcid.org/0000-0003-1156-4154

Table S1: List of mitochondrial haplotypes carried by each individual sorted by region and cohort. Regional codes include Bay of Biscay (BIS), Black Sea (BS), Iberia (IB), Mauritania (MAU), North Atlantic north of the English Channel (NAT). The two cohorts include the (1) 1990s [1990-2002] and (2) 2010s (2012-2015).

Sample ID	Cohort	Region	Haplotype	Source	NCBI Accession ID
FR10001003	1	BIS	Hap_32	Fontaine et al. (2014)	MW732553
FR10002054	1	BIS	Hap_32	Fontaine et al. (2014)	MW732553
FR10003091	1	BIS	Hap_33	Fontaine et al. (2014)	MW732515
FR10003094	1	BIS	Hap_30	Fontaine et al. (2014)	MW732548
FR10007111	1	BIS	Hap_34	Fontaine et al. (2014)	MW732549
FR10205213	1	BIS	Hap_35	Fontaine et al. (2014)	MW732560
FR10304042	1	BIS	Hap_36	Fontaine et al. (2014)	MW732535
FR10402044	1	BIS	Hap_37	Fontaine et al. (2014)	MW732532
FR10403052	1	BIS	Hap_15	Fontaine et al. (2014)	MW732573
FR10405060	1	BIS	Hap_15	Fontaine et al. (2014)	MW732573
FR9712124	1	BIS	Hap_4	Fontaine et al. (2014)	MW732534
FR9903024	1	BIS	Hap_30	Fontaine et al. (2014)	MW732548
FR9904033	1	BIS	Hap_31	Fontaine et al. (2014)	MW732500
FR9904040	1	BIS	Hap_15	Fontaine et al. (2014)	MW732573
B11	1	BS	Hap_44	Fontaine et al. (2014)	MW732521
TK10	1	BS	Hap_46	Fontaine et al. (2014)	MW732532
TK11	1	BS	Hap_47	Fontaine et al. (2014)	MW732550
TK12	1	BS	Hap_48	Fontaine et al. (2014)	MW732525
TK3	1	BS	Hap_45	Fontaine et al. (2014)	MW732524
U30	1	BS	Hap_49	Fontaine et al. (2014)	MW732526
U31	1	BS	Hap_50	Fontaine et al. (2014)	MW732527
U49	1	BS	Hap_51	Fontaine et al. (2014)	MW732551
U64	1	BS	Hap_52	Fontaine et al. (2014)	MW732528
U69	1	BS	Hap_53	Fontaine et al. (2014)	MW732529
U75	1	BS	Hap_50	Fontaine et al. (2014)	MW732527
U93	1	BS	Hap_50	Fontaine et al. (2014)	MW732527
BS51	1	IB	Hap_4	Fontaine et al. (2014)	MW732534
PP04-2002	1	IB	Hap_4	Fontaine et al. (2014)	MW732534
PP118-2004	1	IB	Hap_4	Fontaine et al. (2014)	MW732534
PP29-2002	1	IB	Hap_4	Fontaine et al. (2014)	MW732534
PP30-2002	1	IB	Hap_38	Fontaine et al. (2014)	MW732538
PP63-2002	1	IB	Hap_8	Fontaine et al. (2014)	MW732539
PP68-2002	1	IB	Hap_7	Fontaine et al. (2014)	MW732540
PP79-2000	1	IB	Hap_39	Fontaine et al. (2014)	MW732541
PP79-2003	1	IB	Hap_40	Fontaine et al. (2014)	MW732523
PPH-002	1	IB	Hap_1	Fontaine et al. (2014)	MW732499

PPH009	1	IB	Hap_41	Fontaine et al. (2014)	MW732543
PPH017	1	IB	Hap_42	Fontaine et al. (2014)	MW732545
PPH021	1	IB	Hap_4	Fontaine et al. (2014)	MW732534
PPH027	1	IB	Hap_4	Fontaine et al. (2014)	MW732534
PPH028	1	IB	Hap_4	Fontaine et al. (2014)	MW732534
PPH029	1	IB	Hap_4	Fontaine et al. (2014)	MW732534
PPH030	1	IB	Hap_4	Fontaine et al. (2014)	MW732534
PPH032	1	IB	Hap_4	Fontaine et al. (2014)	MW732534
PPH035	1	IB	Hap_43	Fontaine et al. (2014)	MW732546
PP-156-2012	2	IB	Hap_4	This study	MW732534
PP-175-2012	2	IB	Hap_4	This study	MW732534
PP-177-2014	2	IB	Hap_4	This study	MW732534
PP-179-2014	2	IB	Hap_4	This study	MW732534
PP-183-2013	2	IB	Hap_4	This study	MW732534
PP-185-2013	2	IB	Hap_3	This study	OR557269
PP-187-2013	2	IB	Hap_4	This study	MW732534
PP-194-2013	2	IB	Hap_4	This study	MW732534
PP-197-2013	2	IB	Hap_4	This study	MW732534
PP-198-2013	2	IB	Hap_8	This study	MW732539
PP-21-2015	2	IB	Hap_10	This study	OR557273
PP-217-2013	2	IB	Hap_8	This study	MW732539
PP-218-2013	2	IB	Hap_2	This study	OR557268
PP-227-2014	2	IB	Hap_4	This study	MW732534
PP-234-2013	2	IB	Hap_9	This study	OR557272
PP-236-2013	2	IB	Hap_3	This study	OR557269
PP-242-2013	2	IB	Hap_7	This study	MW732540
PP-246-2013	2	IB	Hap_4	This study	MW732534
PP-25-2015	2	IB	Hap_10	This study	OR557273
PP-255-2013	2	IB	Hap_8	This study	MW732539
PP-266-2012	2	IB	Hap_4	This study	MW732534
PPH-152-2015	2	IB	Hap_11	This study	OR557274
PPH-191-2015	2	IB	Hap_7	This study	MW732540
PPH-199-2014	2	IB	Hap_6	This study	OR557271
PPH-200-2014	2	IB	Hap_6	This study	OR557271
PPH-205-2014	2	IB	Hap_4	This study	MW732534
PPH-207-2014	2	IB	Hap_4	This study	MW732534
PPH-213-2015	2	IB	Hap_4	This study	MW732534
PPH-214-2015	2	IB	Hap_4	This study	MW732534
PPH-215-2015	2	IB	Hap_4	This study	MW732534
PPH-216-2014	2	IB	Hap_4	This study	MW732534
PPH-216-2015	2	IB	Hap_3	This study	OR557269
PPH-217-2015	2	IB	Hap_3	This study	OR557269

PPH-219-2015	2	IB	Hap_4	This study	MW732534
PPH-220-2015A	2	IB	Hap_7	This study	MW732540
PPH-220-2015B	2	IB	Hap_12	This study	OR557275
PPH-221-2015	2	IB	Hap_3	This study	OR557269
PPH-230-2015	2	IB	Hap_4	This study	MW732534
PPH-235-2014	2	IB	Hap_4	This study	MW732534
PPH-254-2014	2	IB	Hap_4	This study	MW732534
PPH-258-2014	2	IB	Hap_5	This study	OR557270
PPH-270-2014	2	IB	Hap_4	This study	MW732534
PPH-271-2013	2	IB	Hap_4	This study	MW732534
PPH-277-2014	2	IB	Hap_4	This study	MW732534
PPH-281-2014	2	IB	Hap_4	This study	MW732534
PPH-282-2014	2	IB	Hap_3	This study	OR557269
PPH-285-2014	2	IB	Hap_4	This study	MW732534
PPH-296-2014	2	IB	Hap_4	This study	MW732534
PPH-297-2014	2	IB	Hap_3	This study	OR557269
PPH-358-2014	2	IB	Hap_4	This study	MW732534
PP-311-2011	2	IB	Hap_2	This study	OR557268
88	1	MAU	Hap_54	Fontaine et al. (2014)	MW732516
89	1	MAU	Hap_54	Fontaine et al. (2014)	MW732516
97	1	MAU	Hap_55	Fontaine et al. (2014)	MW732530
99	1	MAU	Hap_56	Fontaine et al. (2014)	MW732517
100	1	MAU	Hap_60	Fontaine et al. (2014)	MW732519
106	1	MAU	Hap_58	Fontaine et al. (2014)	MW732518
109	1	MAU	Hap_57	Fontaine et al. (2014)	MW732520
125	1	MAU	Hap_57	Fontaine et al. (2014)	MW732520
132	1	MAU	Hap_58	Fontaine et al. (2014)	MW732518
140	1	MAU	Hap_59	Fontaine et al. (2014)	MW732531
141	1	MAU	Hap_60	Fontaine et al. (2014)	MW732519
153	1	MAU	Hap_56	Fontaine et al. (2014)	MW732517
156	1	MAU	Hap_54	Fontaine et al. (2014)	MW732516
189	1	MAU	Hap_57	Fontaine et al. (2014)	MW732520
2000-03	1	NAT	Hap_17	Fontaine et al. (2014)	MW732575
2000-10	1	NAT	Hap_19	Fontaine et al. (2014)	MW732561
2000-24	1	NAT	Hap_20	Fontaine et al. (2014)	MW732572
2000-38	1	NAT	Hap_23	Fontaine et al. (2014)	MW732576
2000-41	1	NAT	Hap_24	Fontaine et al. (2014)	MW732595
2000-44	1	NAT	Hap_25	Fontaine et al. (2014)	MW732579
2000-50	1	NAT	Hap_26	Fontaine et al. (2014)	MW732578
99-24	1	NAT	Hap_16	Fontaine et al. (2014)	MW732574
AK31	1	NAT	Hap_19	Fontaine et al. (2014)	MW732561
IFR1	1	NAT	Hap_27	Fontaine et al. (2014)	MW732556

IFR10	1	NAT	Hap_29	Fontaine et al. (2014)	MW732599
IFR3	1	NAT	Hap_27	Fontaine et al. (2014)	MW732556
IFR4	1	NAT	Hap_27	Fontaine et al. (2014)	MW732556
IFR5	1	NAT	Hap_28	Fontaine et al. (2014)	MW732566
IFR6	1	NAT	Hap_28	Fontaine et al. (2014)	MW732566
IFR8	1	NAT	Hap_27	Fontaine et al. (2014)	MW732556
SV226	1	NAT	Hap_14	Fontaine et al. (2014)	MW732510
VF-05-99	1	NAT	Hap_13	Fontaine et al. (2014)	MW732555
2000-04	1	NAT	Hap_18	Fontaine et al. (2014)	MW732587
2000-26	1	NAT	Hap_21	Fontaine et al. (2014)	MW732588
2000-32	1	NAT	Hap_22	Fontaine et al. (2014)	MW732575
99-22	1	NAT	Hap_15	Fontaine et al. (2014)	MW732573
FRPP4	1	NAT	Hap_4	Fontaine et al. (2014)	MW732534

Table S2: Distribution of the demographic and mutational priors of each parameter of the models used for the simulations in *fastsimcoal* in the ABC-RF analysis.

Parameter	Prior
N1	UN~[1000, 10000]
N2	UN~[10, 100]
Resize	UN~[100, 1000]
Nanc1 = N1/Resize	-
Nanc2 = N2*Resize	-
Nanc3 = Nanc2/Resize	-
Mutation model	*DNA with finite site model*
Recombination	*0 (no recombination)*
Transition rate	*0.33 (no transition bias)*
Mutation rate (μ) per bp, and per gen.	UN~[1 x 10^{-7}, 1 x 10^{-5}]

UN: Uniform distribution [min , max range]

Table S3: List of summary statistics from *Arlsumstat v3.5.2* used in the ABC-RF analysis

Acronym	Summary statistics
gr_K	Number of total haplotypes for both cohorts combined
gr_H	Expected number of haplotypes for both cohorts combined
gr_Hsd	Standard deviation of the expected number of haplotypes
gr_S	Total number of segregating sites for both cohorts combined
gr_sd_S	Standard deviation of the number of segregating sites for both cohorts combined
gr_D	Tajima D for all both cohorts combined
gr_sd_D	Standard deviation of Tajima D for both cohorts combined
gr_FS	Fu's Fs for both cohorts combined
sd_FS	Standard deviation of Fu's Fs for both cohorts combined
gr_Pi	Mean number of pairwise differences for both cohorts combined
gr_sd_Pi	Standard deviation of the pairwise differences for both cohorts combined
K_1	Number of haplotypes in cohort 1
K_2	Number of haplotypes in cohort 2
mean_K	Mean number of haplotypes over cohorts
sd_K	Standard deviation over cohorts of the number of haplotypes
H_1	Expected number of haplotypes in cohort 1
H_2	Expected number of haplotypes in cohort 2
Hsd_1	Standard deviation of the expected number of haplotypes in cohort 1
Hsd_2	Standard deviation of the expected number of haplotypes in cohort 2
mean_H	Mean expected number of haplotypes over cohorts
sd_H	Standard deviation over cohorts of the expected number of haplotypes
S_1	Number of segregating sites in cohort 1
S_2	Number of segregating sites in cohort 2
prS_1	Number of private segregating sites in cohort 1
prS_2	Number of private segregating sites in cohort 2
mean_S	Mean number of segregating sites over cohorts
sd_S	Standard deviation over cohorts of the number of segregating sites
Theta_S	Watterson's estimator of theta
D_1	Tajima D in cohort 1
D_2	Tajima D in cohort 2
mean_D	Mean value of Tajima D over cohorts
sd_D	Standard deviation over cohorts of Tajima D
FS_1	Fu's Fs in cohort 1

FS_2	Fu's Fs in cohort 2
mean_FS	Mean value of Fu's Fs over cohorts
sd_FS	Standard deviation over cohorts of Fu's Fs
Pi_1	Nucleotide diversity in cohort 1
Pi_2	Nucleotide diversity in cohort 2
mean_Pi	Mean nucleotide diversity over cohorts
sd_Pi	Standard deviation over cohorts of nucleotide diversity
Theta_Pi	Tajima's estimator of theta
Theta_K	Ewens' estimator of theta
FST	Pairwise genetic distance between population (Slatkin 1995)
Exp_all	Mean expected frequency of polymorphic sites for both cohorts combined
Exp_all1	Mean expected frequency of polymorphic sites in cohort 1
Exp_all2	Mean expected frequency of polymorphic sites in cohort 2
Obs_all	Mean observed frequency of polymorphic sites for both cohorts combined
Obs_all1	Mean observed frequency of polymorphic sites in cohort 1
Obs_all2	Mean observed frequency of polymorphic sites in cohort 2
Transi	Number of transitions for both cohorts combined
Transi1	Number of transitions in cohort 1
Transi2	Number of transitions in cohort 2
Transv	Number of tranversions for both cohorts combined
Transv1	Number of tranversions in cohort 1
Transv2	Number of tranversions in cohort 2

Table S4. Pairwise differentiation between geographical regions. Above the diagonal, pairwise Hudson's estimator of F_{ST} (Hudson et al. 1992). Below the diagonal, pairwise Φ_{ST} values (Excoffier et al. 1992). *p-value < 0.05; ** p-value < 0.01; *** p-value < 0.001; NS: Not Significant.

	**BS**	**MA**	**IB_O**	**IB_N**	**BB**	**NAT**
**BS**	-	0.88***	0.91***	0.92***	0.53***	0.64***
**MA**	0.88***	-	0.70***	0.70***	0.42***	0.65***
**IB_O**	0.91***	0.70***	-	**-0.08**NS	0.39***	0.62***
**IB_N**.	0.91***	0.68***	**-0.01**NS	-	0.44***	0.74***
**BB**	0.56***	0.41***	0.40***	0.53***	-	0.17*
**NAT**	0.61***	0.62***	0.66***	0.74***	0.12**	-

Table S5: Goodness-of-fit analysis of the ABC-RF analysis. The summary statistics for which the observed values fall outside the confidence interval of the distributions generated from the simulated data sets were considered at outliers. The number of outlying statistics is provided at the 0.1%, 1% and 5% threshold *p*-level. Below are the median values for each outlying statistic simulated under each scenario. The last column shows the observed value for each outlying statistic.

	SC1	SC2	SC3	SC4	SC5	SC6	Obs
**p < 0.05**	0	2	0	5	3	**0**	-
**p < 0.01**	1	1	2	0	0	**0**	-
**p < 0.001**	3	0	2	0	0	**0**	-
**#Outliers**	4	3	4	5	3	**0**	
**gr_K**	-	9.24*	-	26.85*	-	-	20.00
**gr_H**	-	0.22*	-	0.95*	-	-	0.71
**gr_Hsd**	0.005***	-	-	-	-	-	0.06
**gr_S**	-	-	43.00**	-	-	-	17.00
**gr_D**	-	-	0.11***	-	-	-	-1.60
**gr_FS**	-	-	0.41***	-	-	-	-14.48
**K_1**	-	-	-	15.00*	-	-	13
**K_2**	-	-	-	13.73*	-	-	10
**mean_K**	-	-	-	14.37*	-	-	11.5
**gr_Hsd_1**	-	-	-	-	0.03*	-	0.06
**Hsd_1**	0.01***	-	-	-	0.04*	-	0.06
**Hsd_2**	0.02**	-	-	-	0.06*	-	0.11
**sd_H**	0.01***	-	-	-	-	-	0.02
**Exp_all**	-	-	17.21**	-	-	-	6.91
**Transi1**	-	3.76**	-	-	-	-	12

The meaning of the summary statistics acronyms is provided in Table S3.

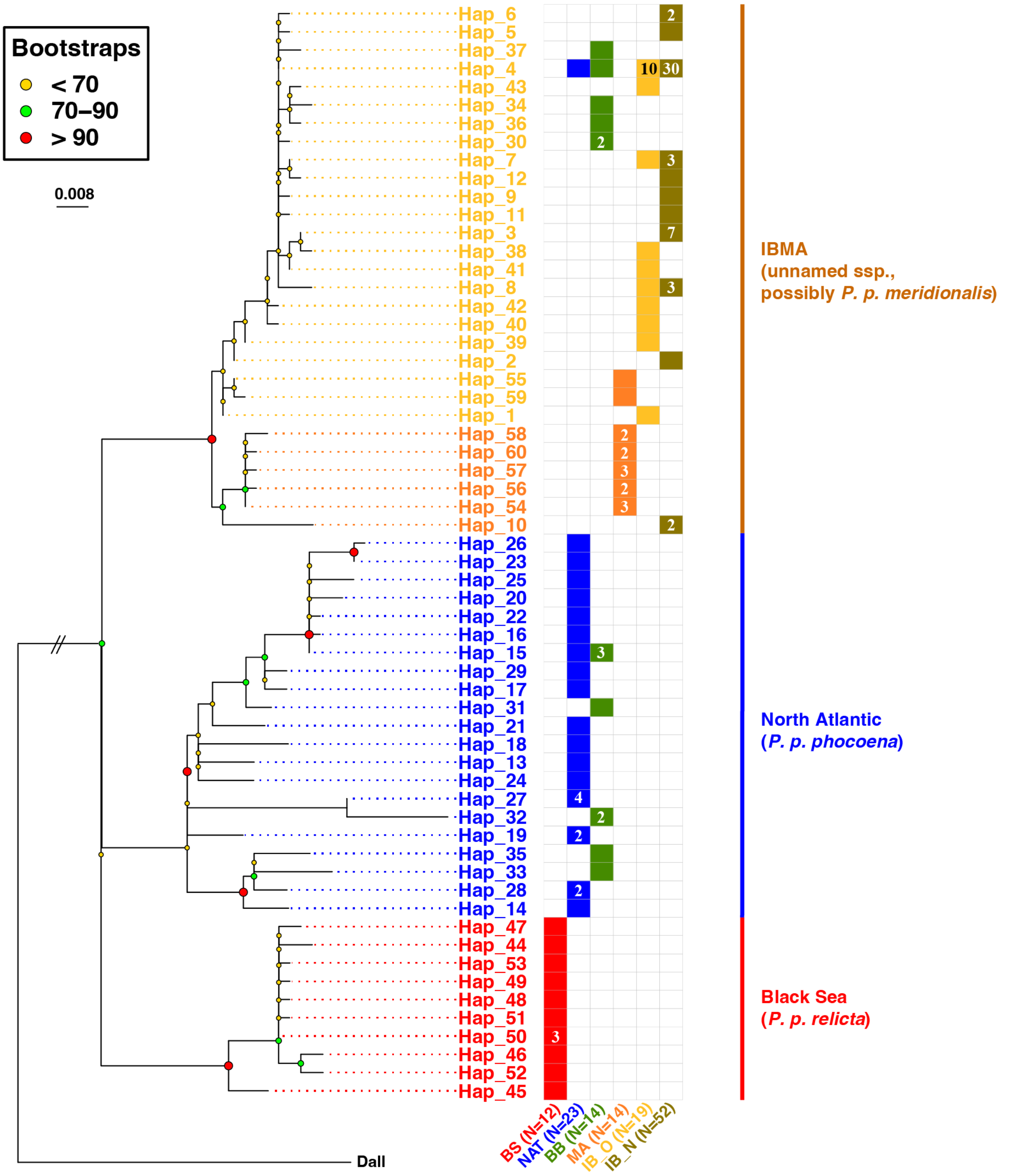

Figure S1. Maximum-likelihood mitochondrial phylogeny among unique mitochondrial haplotypes. Note that some inter-branch length values are extremely small and may lead to difficulty in observing the position of some nodes and their branching patterns. A cladogram with a branch length transformation proportional to the number of tips under the node (as implemented in *FigTree v.1.4.4*) is provided in Figure 3. The color-coded labels show the geographic origin of the haplotype. The numbers within the boxes refer to the number of individuals carrying the haplotype. No number in a box means that the haplotype was observed only once. Population code are as follow: BS=Black Sea (N=12)*; NAT=North Atlantic (N=23)*; BB=Bay of Biscay (N=14)*; MA=Mauritania (N=14)*; IB_O=Iberian Old (N=19)*; IB_N= Iberian new (N=52)**. [* Fontaine et al. 2014; ** This study].

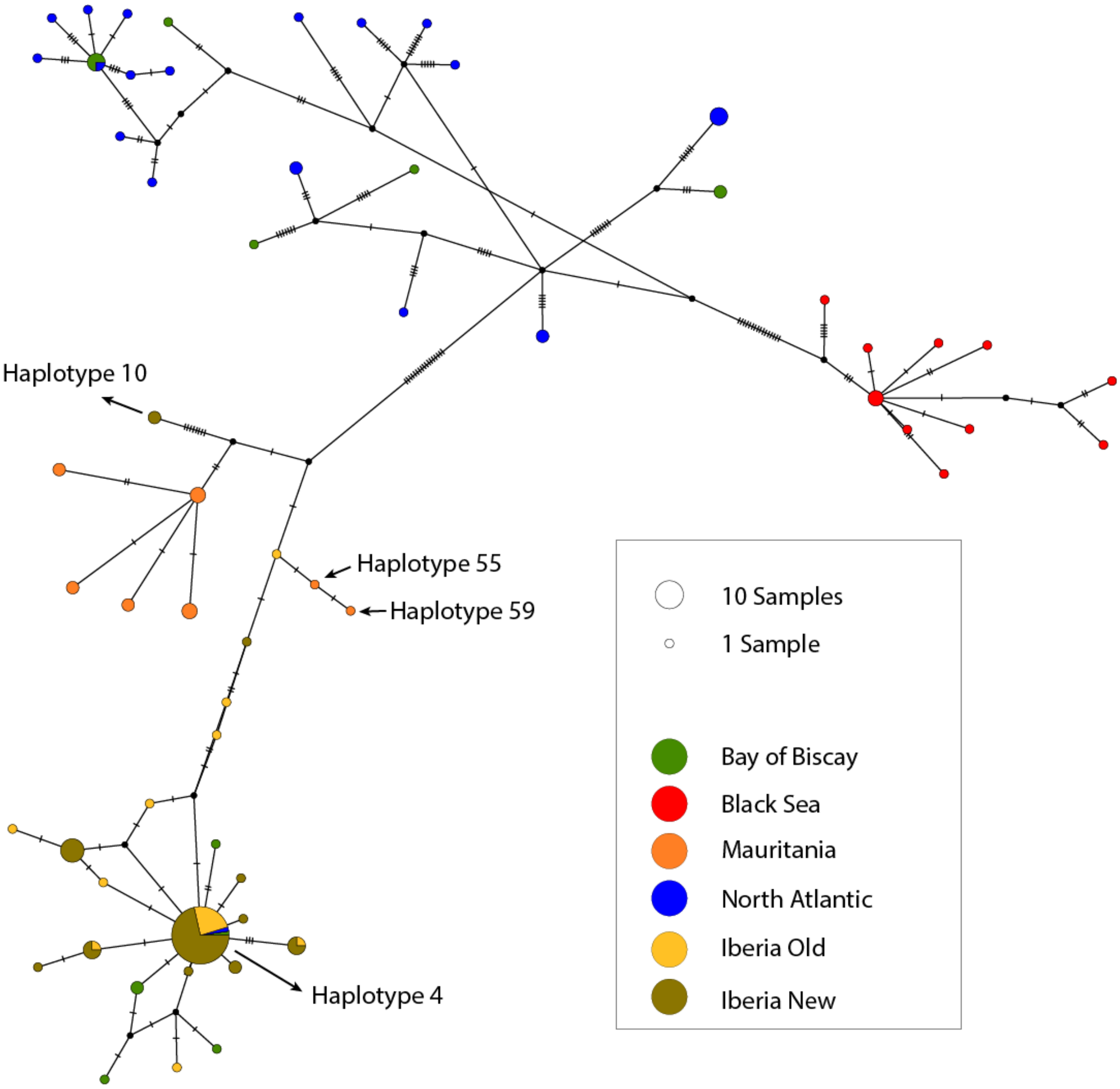

Figure S2. Mitochondrial median-joining haplotype network. Each circle represents a haplotype. The size of each circle is proportional to the haplotype frequency observed in the total sampling, and each pie slice is proportional to the number of each haplotype observed per geographic location. Each dash represents a unique mutational step between haplotypes. A black dot represents a hypothetically unsampled or extinct node. The arrows highlight haplotypes 4 and 10 discussed in the main text.

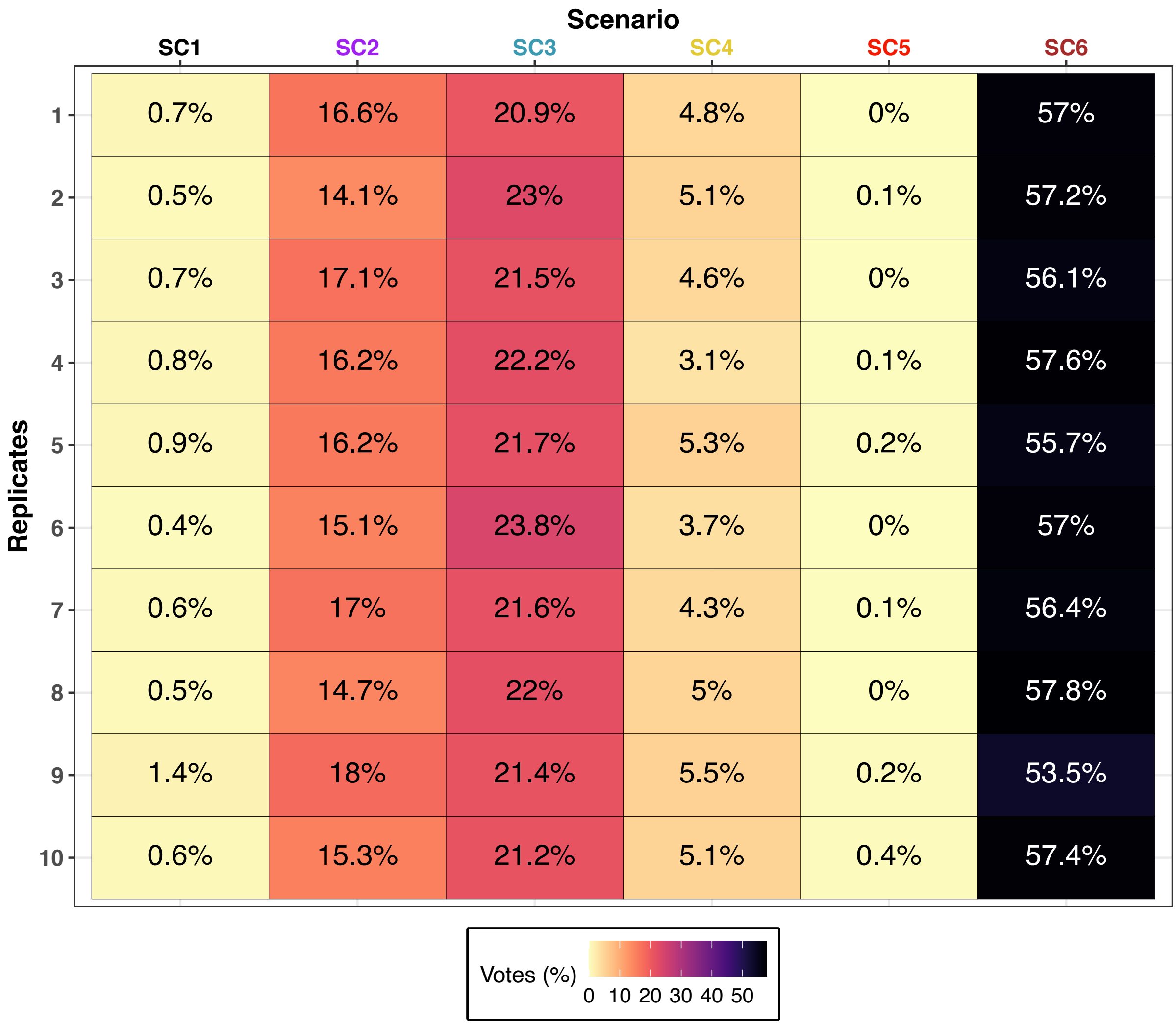

Figure S3. Proportion of ABC-RF classification votes, based on 1,000 decision trees. Each row represents one of the 10 replicates performed.

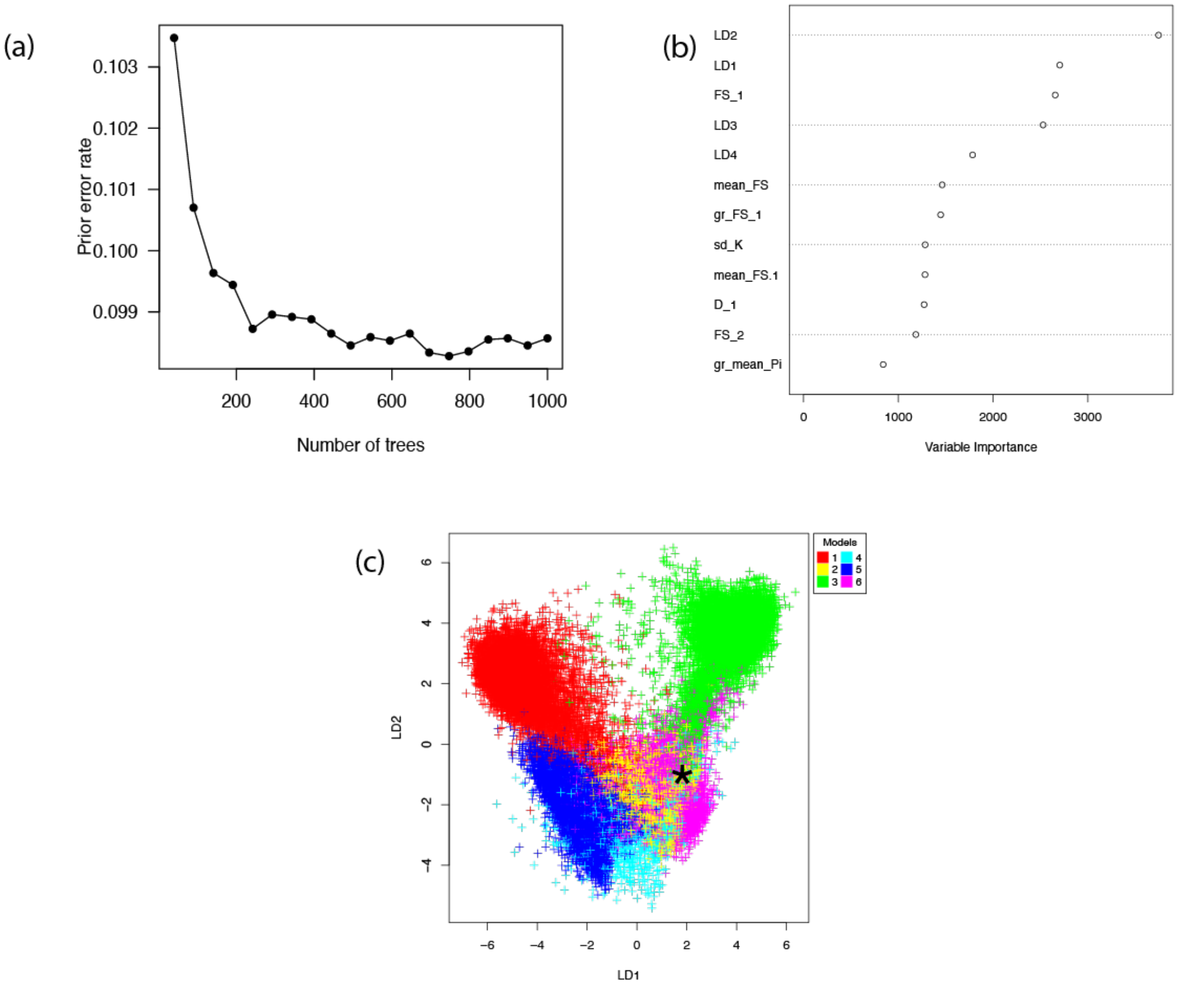

Figure S4. Performance of the ABC-RF in selecting the best model. (A) Evolution of the ABC-RF *prior* error rate with the number of trees in the forest. (B) Contributions of the 12 most important statistics to the Random Forest to discriminate among the competing scenarios. The meaning of the acronym is provided in Table S3. (C) Scatter plot of the first two axes of the Linear Discriminant Analysis displaying the summary statistics obtained from the simulated (+) and observed (✻) data.

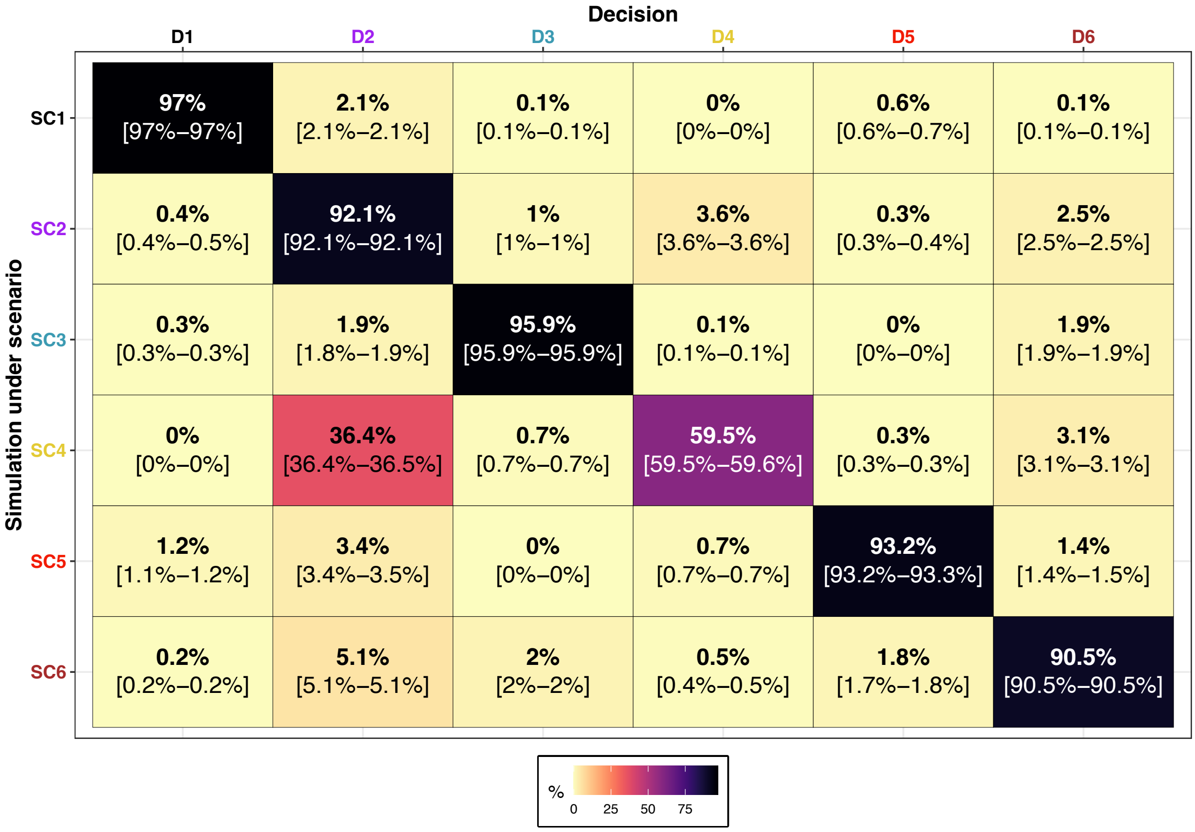

Figure S5. ABC-RF out-of-bag confusion matrix. The diagonal represents the proportion of simulated datasets correctly classified for each demographic scenario. Each row corresponds to the scenario under which simulations were generated and each column the best supported scenario selected by the Random forest classifier (Decision). The values in bold represent the mean proportions over 10 replicates. The brackets show the minimum and maximum values.

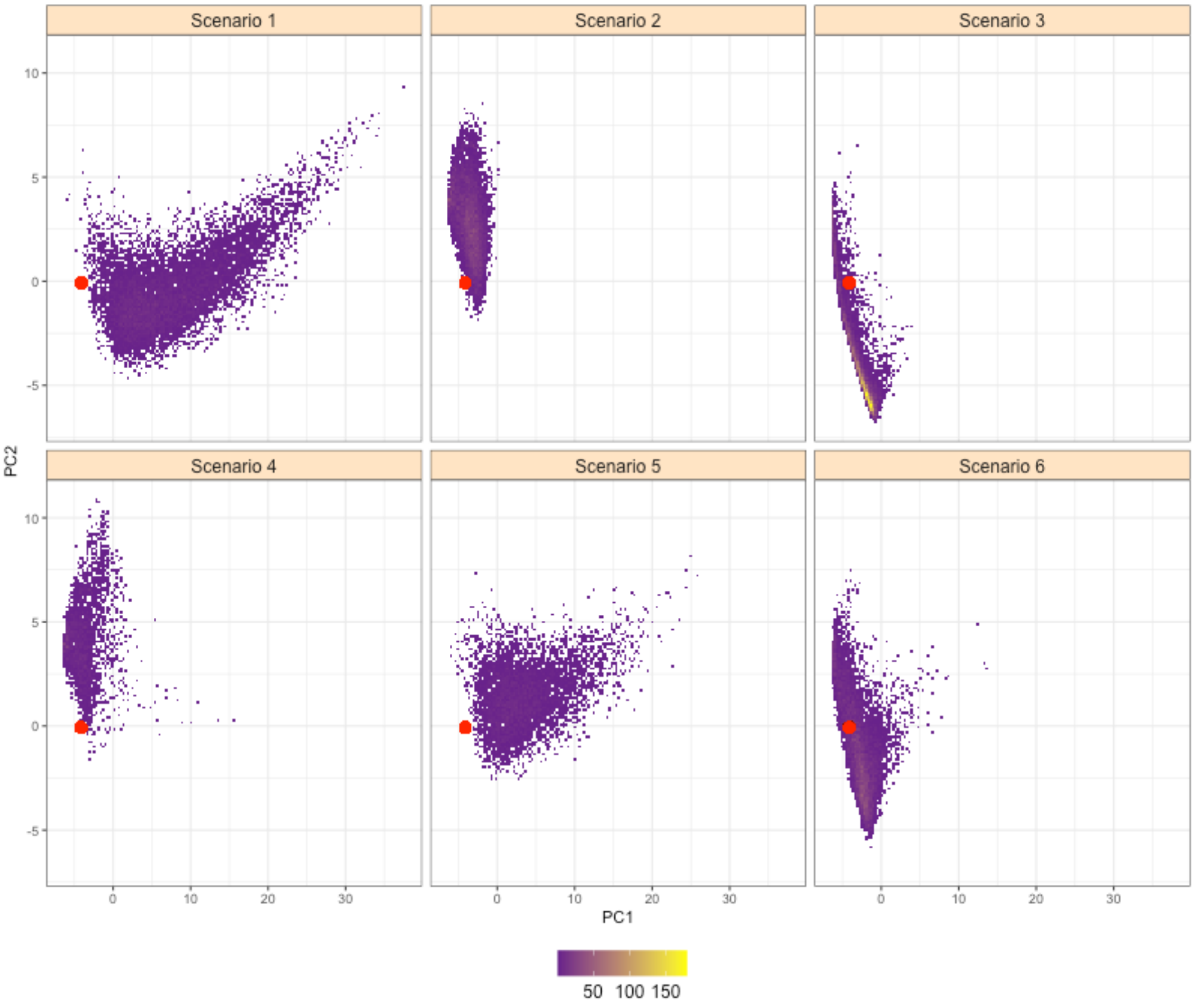

Figure S6. Scatter plot of the summary statistics obtained for the 10k simulations under each demographic scenario in the 2-dimensional space defined by first two principal components (PCs) of the PCA. The observed data set is projected onto each PCA of each scenario as a red dot. Only Scenario 6 is capable of generating simulated values for each summary statistics surrounding those of the observed data.
